# Supplementary figures and images for: Targeted nanodiamonds for identification of subcellular protein assemblies in mammalian cells
Source: PLoS One. 2017 Jun 21;12(6):e0179295. doi: 10.1371/journal.pone.0179295 (PMC5479563; doi:10.1371/journal.pone.0179295)

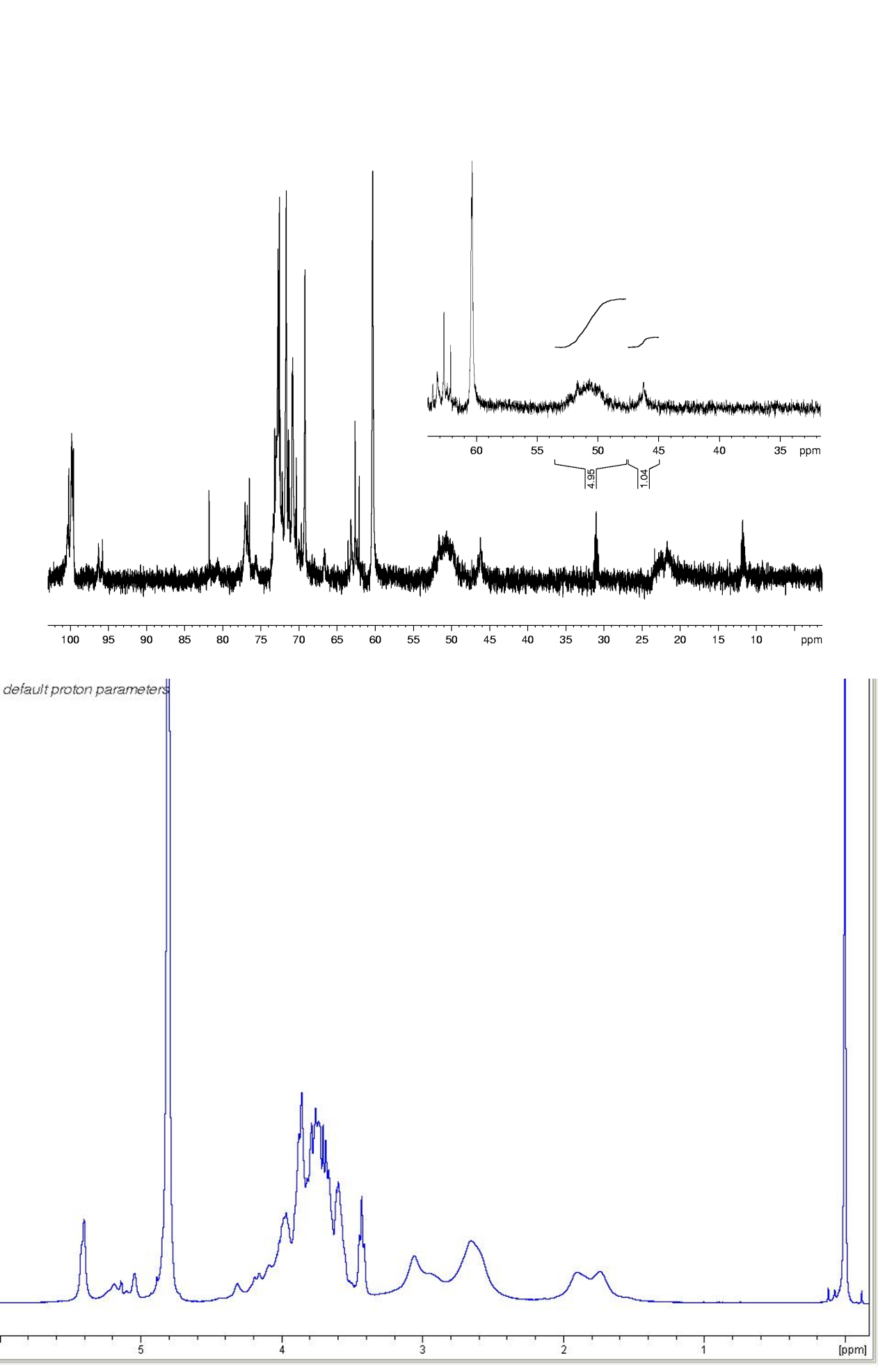

Supplement: S1 Fig — 13C (top) and 1H (bottom) spectrum of maltotriose-conjugated PPI dendrimers recorded on Bruker AVX-500 and DRX-500 NMR systems, respectively. Quantitative comparison of NMR peaks from the 13C spectrum indicates 83% conjugation of PPI dendrimer free amines (—H2C–NH2) with maltotriose (—H2C–NH2–R, R = maltotriose). (PNG) [file pone.0179295.s001.png]

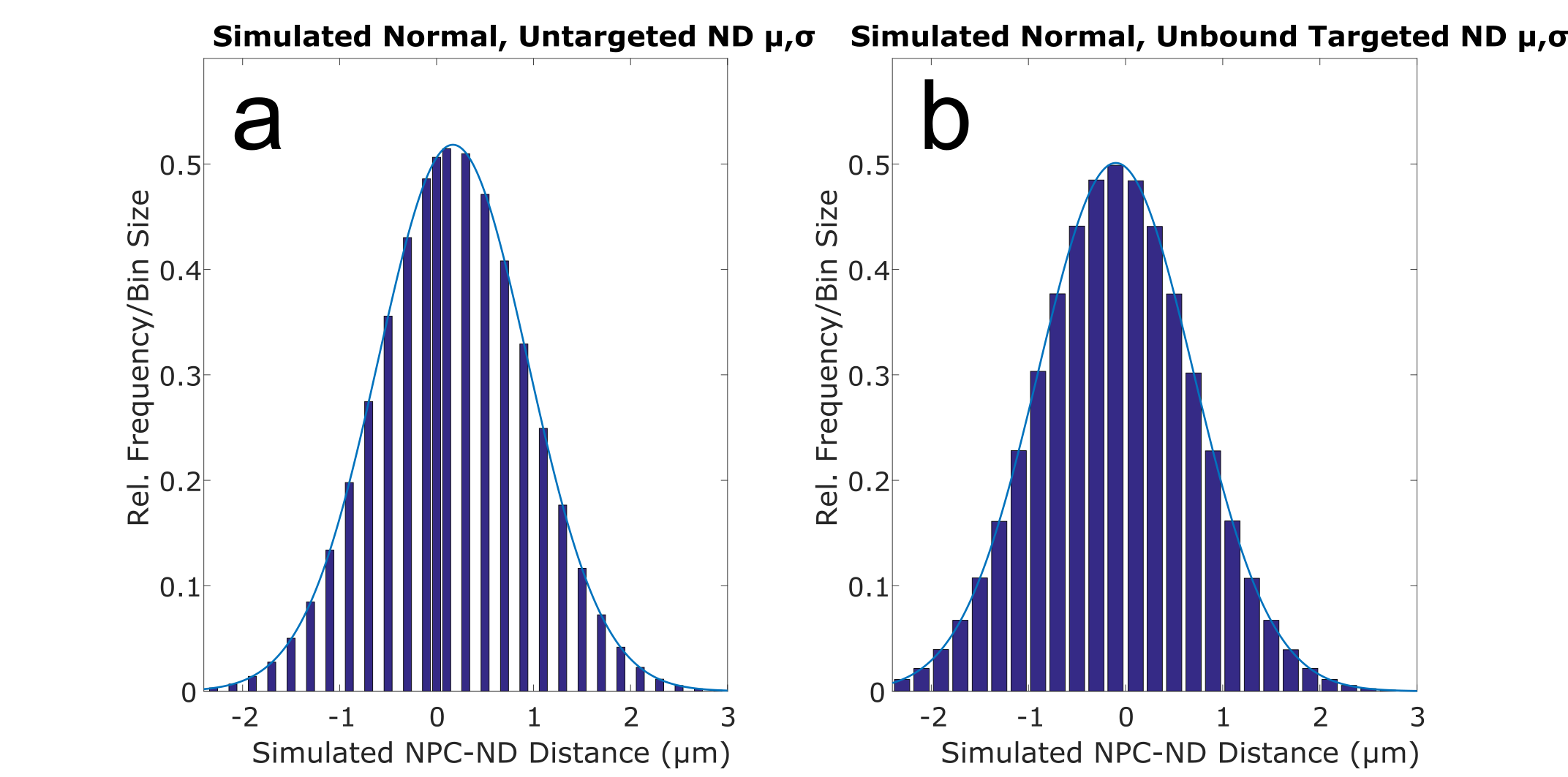

Supplement: S2 Fig — (a) Histogram of simulated counts to confirm that the relative frequency/bin size reproduces the normal distribution for the untargeted ND data. Data was simulated by generating a 10,000,000 element random data set from the normal distribution fit to the untargeted ND data set (μ = 0.168 μm, σ = .770), then binned identically. Bin edges are -2.4, -2.2, -2, -1.8, -1.6, -1.4, -1.2, -1, -0.8, -0.6, -0.4, -0.2, -0.0075, 0.0075, 0.2, 0.4, 0.6, 0.8, 1, 1.2, 1.4, 1.6, 1.8, 2, 2.2, 2.4, 2.6, 2.8, 3. Counts were then divided by 10,000,000 and this relative frequency was divided by the bin width. (b) Histogram of simulated counts to confirm that the relative frequency/bin size reproduces the normal distribution for the unbound targeted ND data. Data was simulated by generating a 10,000,000 element random data set from the normal distribution fit to the unbound targeted ND data set (μ = -.101 μm, σ = .796). This data set was the targeted ND distances with the bound fraction removed consisting of 108. All 10,000,000 elements were binned identically to the entire targeted ND data set. Bin edges are -2.4, -2.2, -2, -1.8, -1.6, -1.4, -1.2, -1, -0.8, -0.6, -0.4, -0.2, -0.0075, 0.0075, 0.2, 0.4, 0.6, 0.8, 1, 1.2, 1.4, 1.6, 1.8, 2, 2.2, 2.4, 2.6, 2.8, 3. Counts were then divided by 10,000,000 and the central bin (bound fraction) was discarded. (PNG) [file pone.0179295.s002.png]
